# Effects of Chlorhexidine and Povidone-Iodine on the SARS-CoV-2 Load: A Systematic Review and Meta-analysis

**DOI:** 10.1055/s-0042-1753470

**Published:** 2022-09-08

**Authors:** Faizul Hasan, Hsiao-Yean Chiu, Eisner Salamanca, Edi S. Ridwan, Bayu S. Wiratama, Hendrik S. Budi

**Affiliations:** 1 School of Nursing, College of Nursing, Taipei Medical University, Taipei, Taiwan; 2 School of Dentistry, College of Dentistry, Taipei Medical University, Taipei, Taiwan; 3 School of Nursing, Faculty of Health Sciences, Alma Ata University, Yogyakarta, Indonesia; 4 Department of Epidemiology, Biostatistics and Population Health, Faculty of Medicine, Public Health and Nursing, Universitas Gadjah Mada, Yogyakarta City, Indonesia; 5 Department of Oral Biology, Dental Pharmacology, Faculty of Dental Medicine, Universitas Airlangga, Surabaya, Indonesia

**Keywords:** mouthwash, oral health, viral load, cycle threshold, infectious disease

## Abstract

The efficacy of mouthwash for reducing the viral load in patients with the novel coronavirus disease 2019 (COVID-19) remains unclear. This systematic review and meta-analysis comprehensively examined the effects of chlorhexidine (CHX) and povidone-iodine (PVP-I) on the viral load in patients with COVID-19. We performed methodological analysis, systematic review, and meta-analysis of included studies using the Comprehensive Meta-analysis Software. PubMed, EMBASE, Cochrane Library, and ProQuest were searched from December 1, 2019, to December 2, 2021. In total, we included 10 studies of 1,339 patients with COVID-19. Compared with the control group, both CHX and PVP-I significantly reduced the number of negative reverse-transcription polymerase chain reaction (RT-PCR) results ( *p* <0.001) among COVID-19 patients. The CHX and PVP-I were effective on reducing the number of negative RT-PCR results in COVID-19 patients. Additional studies using adequate randomization methods and larger samples are warned.

## Introduction

The novel coronavirus was first detected in Wuhan, China, in December 2019. 1 This viral infection, which has caused many deaths globally, was named as novel coronavirus disease 2019 (COVID-19) by the World Health Organization. 2 Previously, severe acute respiratory syndrome (SARS) caused an epidemic in China in 2002. 3 4 5 Both COVID-19 and SARS are caused by viral infections in the respiratory tract, and their courses can be fatal. However, the incubation period of COVID-19 (range: 4–12 days) tends to be longer than that of SARS (range: 2–7 days), 6 in addition to differences in the transmission speed and treatment approach. 7

Because of the COVID-19 pandemic, the services offered in dentistry, including patient management before and after procedures, as well as contaminant waste management, must be improved to ensure the safety of dentists, dental assistants, cleaning staff, and patients. 8 9 Patients requiring dental services have a risk of transmitting or contracting the infection. 7 Similarly, exposure to saliva, blood, or aerosols during dental procedures poses high risks to dentists and dental assistants. 10 11 Therefore, mouthwash represents one modality dentists should be offered to the patients to decrease the viral load in the oral cavity.

Mouthwash has frequently been provided by dentists to patients prior to treatment. 8 Chlorhexidine (CHX) and povidone-iodine (PVP-I) are commonly used for oral preprocedural rinsing in dental offices. 12 13 14 15 A recent study using a network meta-analysis revealed that CHX heated to 47°C is the most effective treatment for reducing viral loads in non–COVID-19 patients. 16 Additionally, five systematic reviews have been reported but only three were registered in the International Prospective Register of Systematic Reviews (PROSPERO). 17 18 19 All three studies reported the efficacy of mouthwash in patients with COVID-19. 20 21 However, no meta-analysis has explored the effects of CHX or PVP-I on the viral load, particularly in patients with COVID-19.

Understanding the effectiveness of CHX or PVP-I against SARS-coronavirus-2 (SARS-CoV-2), the causative agent of COVID-19, is important for increasing the safety of dental practice. The findings of this study provide health care providers with available pieces of evidence of mouthwashes abovementioned to viral load reduction during the COVID-19 pandemic. This current systematic review and meta-analysis comprehensively examined the effect of CHX or PVP-I on the viral load in patients with COVID-19.

## Methods

### Data Sources and Searches

This systematic review was performed according to the Preferred Reporting Items for Systematic Reviews and Meta-analyses (PRISMA) guidelines. 22 This study was registered in PROSPERO (no. CRD42021253915). PubMed, EMBASE, Cochrane Library, and ProQuest were searched from December 1, 2019, to December 2, 2021. We use the following keyword combinations: (“oral rinses” OR “mouth rinses” OR “oral mouth rinses” OR “mouth wash” OR “mouthwashes” OR “mouthwash” OR “mouthwashing”) AND (“viral load” OR “viral burden” OR “viral inactivation” OR “virus inactivation”) AND (“COVID-19” OR “SARS-CoV-2” OR “severe acute respiratory syndrome coronavirus 2” OR “coronavirus” OR “COVID 19” OR “coronavirus disease 2019”). Supplementary Table S1 (available in the online version) described the different search strategies. Additionally, comparable studies that met the inclusion criteria were manually searched in each retrieved study.

### Study Selection

We included full-text original studies with an experimental design that reported the effect of CHX or PVP-I on the SARS-CoV-2 load. No language restrictions were applied, and applicable studies were included if English translations were available. Studies reporting the efficacy of mouthwash other than CHX or PVP-I against COVID-19 and those describing topical antiseptic formulations but intended for either only nasal application or as a surface disinfectant were excluded. Opinions, commentaries and review articles and studies reporting the efficacy of topical antiseptic formulations against related coronaviruses but not specifically against SARS-CoV-2 were excluded. Two reviewers (F. H. and E. S.) independently reviewed the titles and abstracts of potentially eligible studies. Any discrepancies were discussed with a third investigator (H.S.B).

### Data Extraction

Two independent reviewers (F. H. and E. S.) extracted data and addressed inconsistencies. We included the following data: (1) characteristics of the included studies (e.g., first author's name and year of publication), (2) demographic characteristics of the patient population (e.g., age and male percentage and number of participants in each group), (3) intervention characteristics (e.g., type, frequency, and duration), and (4) result. Conflicts were resolved through discussion with the corresponding author (H.S.B) until a consensus was reached.

### Descriptions of Outcome Measures

The reported outcomes included the SARS-CoV-2 RNA viral load according to the number of negative reverse-transcription polymerase chain reaction (RT-PCR) results. We also included study that reported the log _10_ reduction value and cycle threshold (Ct) value using reverse-transcription polymerase chain reaction targeting SARS-CoV-2. 23 We attempted to contact the original authors to obtain additional or missing information via e-mail.

### Risk of Bias Assessment

The quality of the included studies was assessed using the criteria recommended in the Cochrane Handbook for Systematic Reviews of Interventions. 24 We employed the Cochrane risk of bias tool 1.0 (RoB 1.0) for the randomized control trials (RCTs). 25 All included studies were analyzed by two independent reviewers (F. H. and E. S.). Any disagreements were resolved during the consensus meeting.

### Data Synthesis and Analysis

All analyses were conducted using the Comprehensive Meta-analysis Software, 2.0 (Biostat, Englewood, New Jersey, United States). We chose a random-effects model over a fixed-effects model because it is more conservative. 26 The effect size (Hedges' *g* ) was calculated using the mean and standard deviation of the differences in outcomes and sample sizes between the experimental and control groups both before and after testing. Hedges' *g* categories with 95% confidence intervals (CIs) were used to determine the magnitude of the effect size as follows: *g* =0.2 to 0.5, small effect; 0.5< *g* ≤ 0.8, moderate effect; and *g* >0.8, large effect. 27 The *Q* -test and *I* ^2^ statistic were used to examine between-study heterogeneity, with *Q* <0.05 and *I* ^2^ >50%, indicating significant heterogeneity. 28 We did perform subgroup analysis of study using CHX or PVP-I compared with control.

## Results

### Search Result and Study Characteristics

**Fig. 1:**
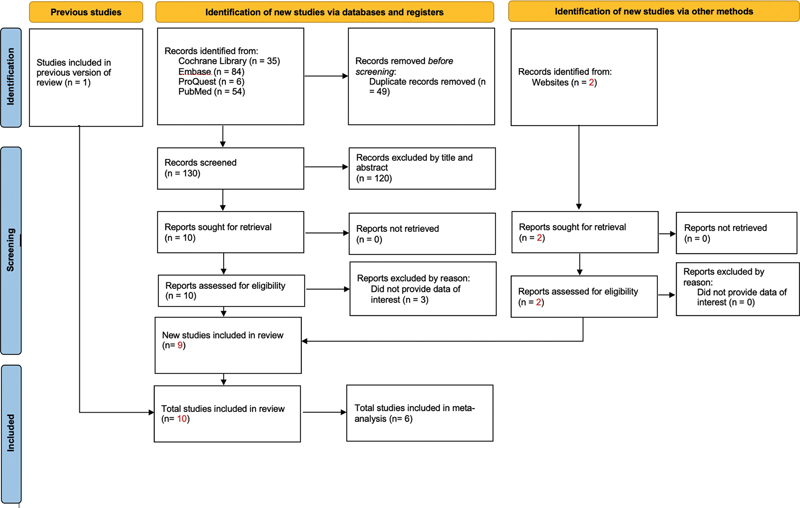
PRISMA 2020 flow diagram for updated systematic reviews which included searches of databases, registers and other sources. PRISMA, Preferred Reporting Items for Systematic Reviews and Meta-analyses.

Fig. 1 presented the electronic search process. We initially identified 179 articles. Among these, 49 duplicate articles and 120 irrelevant studies were excluded. We then retained 10 articles for further analysis. Three studies were excluded because of a lack of relevant data ( Supplementary Table S2 ; available in the online version). We obtained two studies identified via other methods of web searching and one study from previously published articles. In total, we analyzed 10 studies describing the effect of CHX or PVP-I on the SARS-CoV-2 load that were included in the systematic review. 29 30 31 32 33 34 35 36 37 38 Meanwhile, only six studies, reporting the same outcome of interest, were included in the meta-analysis. 30 33 35 36 37 38

Tables 1 and 2 reported the characteristics of the included studies. In this part, the total sample size was 1,339, and the age range was 11 to 90 years.

**Table 1:** Characteristics of participants in the included studies

No.	Author (year)	Country	Study design	Mean age (y)	Male ( *n* )	Sample size ( *n* )	Diagnostic	Study methods
1	Avhad et al (2020) 29	India	RCT	Age range=19–49	NR	40	SARS-CoV-2–positive patients diagnosed by RT-PCR	The participants were given mouthwash containing either CHX 0.2% (study group) or ClO _2_ 0.1% (control group). Patients were told to rinse and gargle with 10mL of undiluted mouthwash three times a day for 7 days (before brushing in the morning, after meals in the afternoon and at night) with daily follow-up
2	Choudhury et al (2021) 30	Bangladesh	RCT	Age range=11–90	484	606	SARS-CoV-2–positive patients diagnosed by RT-PCR within 1 day	The 606 participants were enrolled and randomly allocated to one of two groups after proving consent. In group A, 303 patients received mouthwash/gargle, nose drops and eye drops containing 1% PVP-I 4hours a day for 4 weeks, in addition to any necessary symptomatic treatment. In group B, 303 patients were instructed to use warm water to cleanse their mouth, nasal cavity and eyes 4hours daily for 4 weeks, followed by symptomatic treatment as needed. Every third, fifth, and seventh day, RT-PCR was performed, and thyroid hormone levels were evaluated at the conclusion of the fourth week for follow-up
3	Costa et al (2021) 31	Brazil	RCT	*I* =40.5±13.5 *C* =38.5±11.9	*I* =23 *C* =27	110	Presence of flu-like symptoms for 3 to 7 days, and a positive test for SARS-CoV-2	After the fast antigen test, the test group volunteers gargled for 30 seconds with 0.12% chlorhexidine gluconate, then spat and rinsed for 30 seconds with another 15mL of the test substance. The control group used a placebo. The 15-mL portions of the substances were individually packaged for each volunteer and labelled as solution A or B to prevent professional or volunteer identification. The placebo solution had the same flavor and color as the active ingredient
4	Eduardo et al (2021) 32	Brazil	RCT	CPC+Zn=46 (34–88) HP=62 (40–87) CHX=53.5 (49–88) HP+CHX=53 (40–72) C=59 (36–85)	CPC+Zn=5 HP=4 CHX=7 HP+CHX=10 *C* =6	60	SARS-CoV-2–positive patients diagnosed by RT-PCR using nasal swabs	Patients were instructed to rinse as specified by the manufacturer product and to spit out the solution following rinsing. The following volumes and durations of rinsing were used: a) Placebo group: rinse with 20mL for 1 min b) CPC+Zn group: rinse with 20mL for 30 s c) HP group: rinse with 10mL for 1 min d) CHX group: rinse with 15mL for 30 s e) HP+CHX group: rinse with 10mL of HP mouthwash for 1min, followed by rinsing with 15mL of CHX mouthwash for 30 s
5	Elzein et al (2021) 33	Lebanon	RCT	PVP-I=39.9±14.2 CHX=47±15.4 *C* =57.2±22.5	PVP-I=13 CHX=10 *C* =2	77 (only 61 included in the analysis)	SARS-CoV-2–positive patients diagnosed by RT-PCR using nasopharyngeal swabs within 2 days	Participants were randomly assigned to one of three groups. The same trained operator discussed, performed and supervised COVID-19 infection control sampling in the patient's room. Patients self-sampled in the early morning on an empty stomach and prior to brushing their teeth. Solution A was PVP-I 1%, solution B was chlorhexidine 0.2% and solution C was distilled water as a placebo. To begin, participants were asked to cough out 2mL of saliva from their throats into a sterile container. Then, participants in groups A ( *n* =33), B ( *n* =33) and C ( *n* =11) were instructed to gargle with their assigned treatment for 30 s and then spit out the solution. Saliva collection was repeated after 5min into a second sterile container. Each cup was labelled with the patient's name and the date of saliva collection, and infected trash was properly disposed. Each sample was then placed in separate tubes containing 2mL of viral transport medium and delivered to the COVID-19 unit laboratory at a Lebanese university for PCR processing
6	Guenezan et al (2021) 34	France	RCT	Median *I* =33 (IQR: 23–46) *C* =57 (IQR: 45-68)	*I* =4 *C* =4	24	SARS-CoV-2–positive patients diagnosed by RT-PCR using nasopharyngeal swabs within 48hours	Patients were randomized (1:1) to the control (no intervention, *n* =12) or intervention group ( *n* =12). After an additional nasopharyngeal swab was obtained for viral quantification at baseline, patients used four mouthwashes and gargles containing 25mL of 1% aqueous PVP-I solution (Mylan, Merignac, France), followed by one 2.5-mL nasal pulverization of the same solution using an intranasal mucosal atomization device connected to a 5-mL syringe while sniffing and one application on each nasal mucosa During the initial decolonization session, patients were provided the necessary instructions and supplies and a guide to enable them complete the remaining sessions four times a day for 5 days
7	Huang and Huang (2021) 35	The United States	Randomized, prospective cohort	Median 62 (range: 23-89)	171	294	SARS-CoV-2–positive patients diagnosed by RT-PCR using nasopharyngeal swabs	CHX was provided to the research group to use as an oropharyngeal rinse, but not to the control group. Each patient received a unit dose cup containing 0.5 ounces (15mL) of commercially available CHX (0.12 percent). Patients were subsequently observed to self-administer the solution for 30 s twice daily as a thorough oral rinse. In the second trial group, a CHX spray was added to the oral rinse regimen. Following the patient's usage of CHX as an oral rinse, a provider sprayed three sprays (a total of roughly 1.5mL) of the CHX solution into the posterior oropharynx using a spray applicator. The patient was advised to vocalize "ah" for 5 s, while the solution was sprayed to open the posterior pharynx. This procedure was repeated twice daily for 4 days
8	Mohamed et al (2020) 36	Malaysia	RCT	Range=22–56	16	20	SARS-CoV-2–positive patients diagnosed by RT-PCR within 1 day	Twenty identical envelopes were used to randomly assign patients to one of four arms: gargle with 1% PVP-I (group A), gargle with essential oils (group B), gargle with tap water (group C) or no intervention (group D) • Group A patients were instructed regarding the proper Betadine gargling technique. The patients were advised to gargle with 10mL of Betadine, tilt their heads backwards and gargle for 30 s three times daily for 7 days. • Group B patients were instructed regarding the proper Listerine gargling technique. Patients were asked to gargle for 30 s three times daily for 7 days with 20mL Listerine by tilting their heads backwards • Group C patients received instructions regarding the proper gargling technique using tap water. The patients were told to gargle with 100mL of tap water with their heads bent backwards for 30 s three times daily for 7 days • Group D patients were informed of their participation in this trial. They were handled in accordance with the hospital's usual protocol with no further intervention
9	Mukhtar et al (2021) 37	Qatar	RCT	49.55	82	92	SARS-CoV-2–positive patients diagnosed by RT-PCR using combined nasopharyngeal-oropharyngeal swabs within 1 day	All patients were treated according to prespecified protocols for their respective “categories”" in accordance with Communicable Diseases Center guidelines. Antivirals, antibiotics and steroids were included, as well as hydroxychloroquine and convalescent plasma transfusion (where indicated). Meanwhile, the “intervention” group received gargled with rinses for at least 30 s three times daily in addition to their regular routines. This solution contained 15mL of CHX 0.2% (oral rinse) and 5mL of HP 6% (to make a final concentration of 2%). The contents of the solution were blended at the bedside and administered to the subjects. They were required to wait at least 5min after using the mouthwash before rinsing their mouths with tap water, eating or drinking. Because the underlying hypothesis was to assess regular “repeated usage” over a lengthy period (2 weeks), participants who missed a day or more of intervention usage (>3 doses) were regarded to have withdrew, and they were omitted from the analysis. Initially, patients were recommended to use the mouthwash for 1min (with a maximum of 2min of contact time with the oral cavity), but this was decreased to 30 s because of the difficulty of continuous use given the high oxygen requirements (for all cases)
10	Seneviratne et al (2021) 38	Singapore	RCT	PVP-I=40.7±11.5 CHX=43.6±8.6 CPC=35.7±8.5 *C* =36±14.1	PVP-I=4 CHX=6 CPC=4 *C* =1	16	SARS-CoV-2–positive patients diagnosed by RT-PCR within a mean of 5.55 days	Patients in the PVP-I group used 5mL of PVP-I mouthwash (commercially available as Betadine Gargle and Mouthwash 10mg) diluted with 5mL of water (0.5% w/v), whereas those in the CHX group used 15mL of undiluted CHX mouthwash (commercially available as Pearly White Chlor-Rinse, 0.2% w/v). Patients in the CPC group and the water control group rinsed their mouths with 20mL of CPC 0.075% (commercially available as Colgate Plax mouthwash) and 15mL of sterile water, respectively. Three millilitres of saliva were collected again from all subjects at 5min, 3 and 6hours

Abbreviations: C, control; CHX, chlorhexidine gluconate; ClO _2_ , chlorine dioxide; CPC, cetylpyridinium chloride; HP, hydrogen peroxide; I, intervention; IQR, interquartile range; PVP-I, povidone–iodine; RCT, randomized control trial; RT-PCR, reverse transcription–polymerase chain reaction; SARS-CoV-2, severe acute respiratory syndrome-coronavirus-2; Zn, zinc.

**Table 2:** Characteristics of the included studies

No.	Author (year)	Study group ( *n* )	Control ( *n* )	Intervention/test product	Follow-up time	Result
1	Avhad et al (2020) 29	CHX group ( *n* =20)	ClO _2_ group ( *n* =20)	CHX 0.2%/mouthwash (Guard OR, Group Pharmaceuticals Ltd., India) ClO _2_ 0.1%/mouthwash (Freshclor, Group Pharmaceuticals Ltd., India)	7 d	Baseline: Study group: 20 positives Control group: 20 positives Posttest: Day 8: Study group: 12 positives, 8 negatives Control group: 8 positives, 12 negatives • CHX and ClO _2_ reduced the oral viral load
2	Choudhury et al (2021) 30	PVP-I 1% group ( *n* =303)	Water group ( *n* =303)	PVP-I 1%	3 d 5 d 7 d	Baseline: Study group: 303 positives Control group: 303 positives Post-test: Number of positive RT-PCR results PPV-I 1% group 3 days: 35 positives, 268 negatives 5 days: 24 positives, 279 negatives 7 days: 8 positives, 295 negatives Water group 3 days: 291 positives, 12 negatives 5 days: 268 positives, 35 negatives 7 days: 213 positives, 90 negatives Number of total outcomes PPV-I 1% group Hospitalized: 2 (0.66%) Hospitalized+oxygen support: 10 (3.3%) Death: 2 (0.66%) Water group Hospitalized: 4 (4.62%) Hospitalized+oxygen support: 63 (20.79%) Death: 17 (5.61%) • The use of PVP-I 1% as mouthwash/gargle, nasal spray or ocular drop is simple, quick and cost-effective for reducing COVID-19–related mortality and morbidity
3	Costa et al (2021) 31	CHX group ( *n* =55)	Placebo group ( *n* =55)	15mL CHX 0.12%	5 min 60 min	Baseline: Mean Ct value •CHX group 29.83±4.23 •Placebo group 31.50±4.09 Posttest: 5min: mean Ct value •CHX group 32.02±5.17 •Placebo group 31.10±4.70 60 min: mean Ct value •CHX group 32.28±4.73 •Placebo group 32.26±4.64 •CHX was effective in reducing salivary SARS-CoV-2 load for at least 60minutes
4	Eduardo et al (2021) 32	CPC+Zn group ( *n* =12) HP group ( *n* =12 CHX group ( *n* =12) HP+CHX group ( *n* =12)	Placebo group ( *n* =12)	20mL CPC 0.075%, Zn 0.28% (Colgate Total 12, Colgate-Palmolive Company, Brazil); 10ml 1.5% HP (Peroxyl, Colgate-Palmolive Company, USA) 15mL CHX 0.12% (PerioGard, Colgate- Palmolive Company, Brazil) 10ml HP 1.5%+15mL CHX 0.12% (Peroxyl+PerioGard, Colgate-Palmolive Company)	Posttreatment 30 min 60 min	Baseline: Mean Ct value •CPC+Zn group 27.58±3.12 •HP group 28.21±3.17 •CHX group 25.78±5.34 •HP+CHX group 30.33±3.91 •Placebo group 29.64±1.59 Posttest: Posttreatment: mean Ct value •CPC+Zn group 31.60±4.13 •HP group 31.70±4.71 •CHX group 26.26±5.02 •HP+CHX group 30.75±4.7 •Placebo group 28.95±2.53 30 min: mean Ct value •CPC+Zn group 28.58±5.18 •HP group 29.75±3.6 •CHX group 27.21±5.07 •HP+CHX group 29.85±5.12 •Placebo group 28.32±2.27 60 min: mean Ct value •CPC+Zn group 28.11±6.03 •HP group 30.17±2.27 •CHX group 26.84±4.64 •HP+CHX group 31.23±4.34 •Placebo group 28.58±2.37 Mouthwash containing CPC+Zn, and CHX reduced SARS-CoV-2 viral load in saliva after 60 min after rinsing, while HP mouthwash reduced viral load for 30 min after rinsing
5	Elzein et al (2021) 33	Group A/PVP-I 1% ( *n* =33) Group B/CHX 0.2% ( *n* =33)	Water group ( *n* =11)	PVP-I 1% CHX 0.2%	30 s	Baseline: Mean Ct value Group A: 29.88±6.2 Group B: 27.69±7.16 Group C: 31.53±2.72 Posttest: Mean Ct value Group A: 34.36±6.3 Group B: 33.9±7.08 Group C: 31.47±3.51 Mean difference (pre–post) Group A: 4.45 ( *p* <0.0001) Group B: 5.69 ( *p* <0.0001) Group C: 0.06 ( *p* >0.566) A significant difference in the mean Ct value difference ( *p* <0.0001) between the paired samples in groups A (1% povidone-iodine) and B (0.2% chlorhexidine) No significant difference ( *p* =0.566) existed between before and after the experiment in the control group C (distilled water)
6	Guenezan et al (2021) 34	PVP-I group ( *n* =12)	No intervention group ( *n* =12)	PVP-I	1 d 3 d 5 d 7 d	Baseline: Intervention: mean 5.51±0.61 log _10_ copies/mL Control: mean 5.32±0.92 log _10_ copies/mL Posttest: Day 1 Intervention: mean 4.87±0.89 log _10_ copies/mL Control: mean 5.42±0.57 log _10_ copies/mL Day 3 Intervention: mean 4.28±0.77 log _10_ copies/mL Control: mean 4.53±0.53 log _10_ copies/mL Day 5 Intervention: mean 2.91±0.8 log _10_ copies/mL Control: mean 3.13±1.18 log _10_ copies/mL Day 7 Intervention: mean 2.08±1.2 log _10_ copies/mL Control: mean 2.04±0.85 log _10_ copies/mL · The use of PVP-I 1% aqueous had no effect on the viral RNA level over time.
7	Huang and Huang (2021) 35	CHX group (oropharyngeal rinse; *n* =66) CHX group (oropharyngeal rinse+spray; *n* =93)	Control 1 ( *n* =55) Control 2 ( *n* =80)	15mL CHX 0.12% (oropharyngeal rinse), 1.5mL CHX spray	4 d	Baseline: Study 1: 66 positives Control 1: 55 positives Study 1: 93 positives Control 1: 80 positives Posttest: COVID-19 PCR results for swabs Day 4 •15mL CHX 0.12% (oropharyngeal rinse): 41 negative, 25 positives •Control 1: 3 negatives, 52 positives •15mL CHX 0.12% (oropharyngeal rinse) plus 1.5mL CHX spray: 80 negatives, 13 positives •Control: 5 negatives, 75 positives CHX reduced the oral viral load
8	Mohamed, et al (2020) 36	PVP-I 1% group ( *n* =5) Essential oils group ( *n* =5) Tap water group ( *n* =5)	Control group ( *n* =5)	•PVP-I 1% •Essential oils/Listerine •Tap water	4 d 6 d 12 d	Posttest: COVID-19 PCR results for swabs Day 4 •PVP-I 1%: 5 negative, 0 positive, 0 indeterminate •Essential oils/Listerine: 4 negative, 1 positive, 0 indeterminate •Tap water: 2 negative, 3 positive, 0 indeterminate •Control: 1 negative, 2 positive, 2 indeterminate Day 6 •PVP-I 1%: 5 negative, 0 positive, 0 indeterminate •Essential oils/Listerine: 4 negative, 1 positive, 0 indeterminate •Tap water: 2 negative, 1 positive, 2 indeterminate •Control: 0 negative, 2 positive, 3 indeterminate Day 12 •PVP-I 1%: 5 negative, 0 positive, 0 indeterminate •Essential oils/Listerine: 4 negative, 0 positive, 1 indeterminate •Tap water: 2 negative, 2 positive, 1 indeterminate •Control: 1 negative, 3 positive, 1 indeterminate The viral load differed between the PVP-I 1% group and control group on days 4, 6 and 12 ( *p* =0.048, 0.008, and 0.048, respectively However, the viral load did not differ between the PVP-I 1% and tap water groups, Listerine and control groups and Listerine and tap water groups PVP-I 1% and essential oils displayed significant potential for use in the treatment and management of stage 1 COVID-19
9	Mukhtar et al (2021) 37	CHX+HP group ( *n* =46)	Control group ( *n* =46)	10mL of CHX 2%+5mL of HP 6%	5 d 15 d	Baseline: Day 0 COVID RT-PCR test results •Intervention (n=46): 0 negative, 0 inconclusive •Control (n=46): 0 negative, 0 inconclusive Post-test Day 5 COVID RT-PCR test results •Intervention (n=45): 6 negative, 5 inconclusive •Control (n=44): 0 negative, 6 inconclusive Day 15 COVID RT-PCR test results •Intervention (n=43): 15 negative, 14 inconclusive •Control (n=44): 9 negative, 17 inconclusive Total hospital stays •Intervention: mean=8.11 (95% CI=6.19–10.02) •Control: mean=9.43 (95% CI=7.15–11.72) The regular use of mouthwash in patients hospitalized for COVID-19 seems to improve outcomes, as evidenced by the significantly earlier conversion to a “COVID-negative” status by 5 days of treatment
10	Seneviratne, et al., 2021 38	PVP-I group ( *n* =4) CHX group ( *n* =6) CPC group ( *n* =4)	Water group ( *n* =2)	•5mL of PVP-I (0.5% w/v) •15mL of CHX of undiluted (0.2% w/v) •20mL of CPC 0.075%	5 min 3 h 6h	Baseline: Mean Ct value •PVP-I group 21.97±6.37 •CHX group 29.18±3.47 •CPC group 31.88±2.73 •water group 26.41±1.29 Posttest: 5min: mean Ct value •PVP-I group 24.29±7.61 •CHX group 27.89±3.1 •CPC group 32.35±2.61 •water group 25.32±1.56 3h: mean Ct value •PVP-I group 25.37±6.1 •CHX group 30.36±2.2 •CPC group 30.72±3.32 •water group 23.2±0.81 6h: mean Ct value •PVP-I group 21.71±5.71 •CHX group 27.73±3.12 •CPC group 31.53±3.05 •water group 22.03±1.93 The CPC mouth rinse yielded a significantly increased the Ct value at 5min (1) and 6h (0.9) compared to that in the water group ( *p* <0.05) Similarly, the use of PVP-I increased the Ct value compared to the result in the control group at 6h ( *p* <0.01) The use of CPC and PVP-I formulated commercial mouth rinses may have a sustained effect on reducing the salivary SARS-CoV-2 viral load in patients with COVID-19

Abbreviations: CI, confidence interval; ClO _2_ , chlorine dioxide; CHX, chlorhexidine digluconate; CPC, cetylpyridinium chloride; Ct, cycle threshold; HP, hydrogen peroxide; PVP-I, povidone–iodine; SARS-CoV-2, severe acute respiratory syndrome coronavirus 2; Zn, zinc.

### Effects of Mouthwash on the Number of Negative Reverse-Transcription Polymerase Chain Reaction Results

**Fig. 2:**
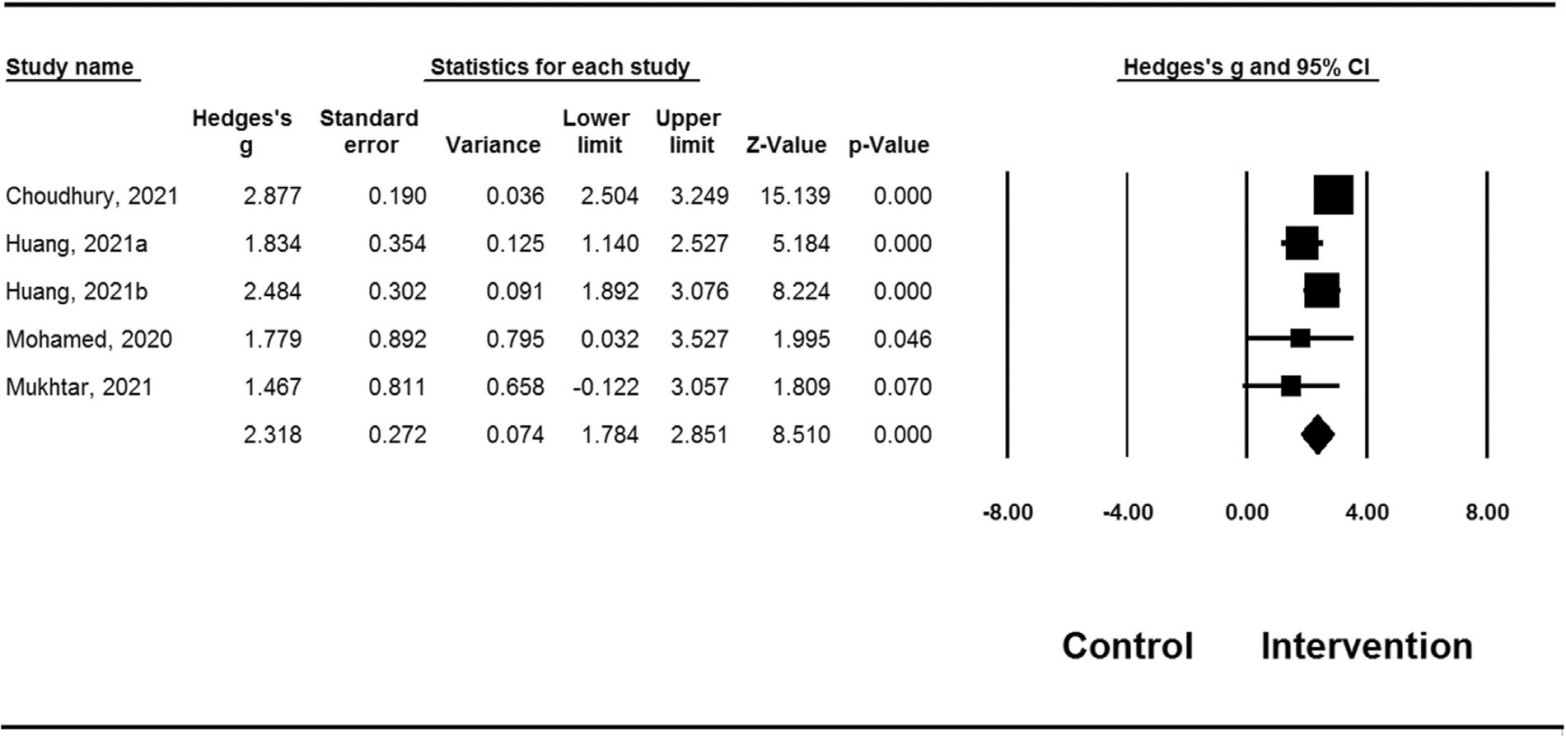
Forest's plot of mean effect sizes for number of negative RT-PCR results between intervention group compare with control group. Heterogeneity: *Q* =9.63, *p* =0.05, *I* ^2^ =58.46. CI, confidence interval; RT-PCR, reverse transcription polymerase chain reaction.

Four studies compared the immediate posttreatment effect of mouthwash (CHX and PVP-I) on the viral load to control in patients with COVID-19 ( Fig. 2 ). The intervention group had a significantly reduced number of negative RT-PCR results compared with the control group (mean effect size of 2.32 [95% CI=1.78–2.85; *p* <0.001]). There was evidence of heterogeneity across these studies ( *Q* =9.63, *p* =0.05, *I* ^2^ =58.46).

**Fig. 3:**
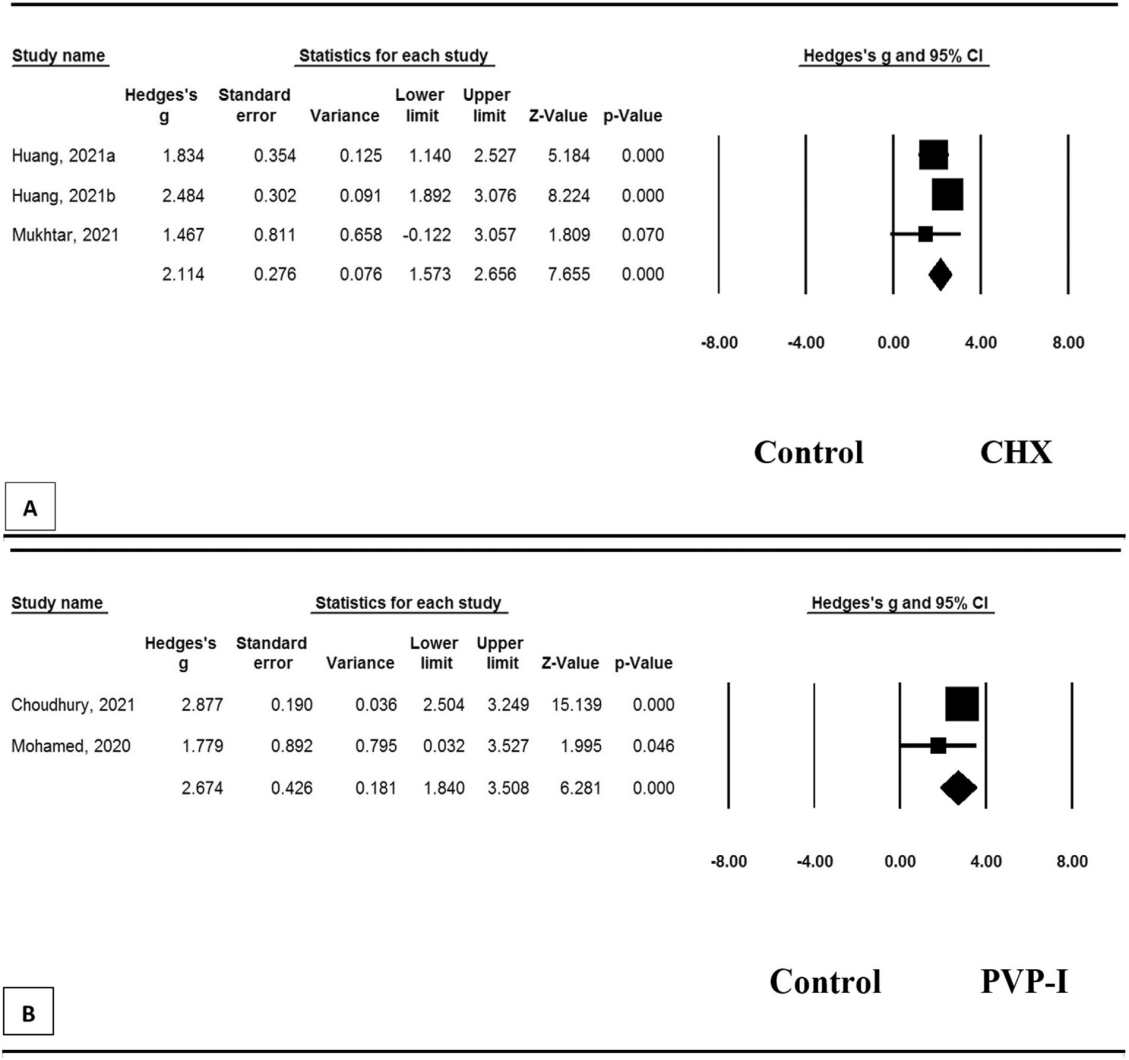
Forest's plot of subgroup mean effect sizes for number of negative RT-PCR results between intervention and control group. ( **A** ) CHX compare with control. ( **B** ) PVP-I compare with control. ( **A** ) Heterogeneity: *Q* =2.73, *p* =0.26, *I* ^2^ =26.71; ( **B** ) heterogeneity: *Q* =1.45, *p* =0.23, *I* ^2^ =30.93. CHX, chlorhexidine; CI, confidence interval; PVP-I, povidone-iodine; RT-PCR, reverse transcription polymerase chain reaction.

We further explore the subgroup analysis of the study using CHX compare with control and using PVP-I compare with control ( Fig. 3A and 3B ). The study used CHX yielded a significant reduction in the number of negative RT-PCR results compare with the control group (mean effect size of 2.11 [95% CI=1.57–2.66; *p* <0.001]; Fig. 3A ). Similarly, the study used PVP-I also significantly reduced the number of negative RT-PCR results compared with the control group (mean effect size of 2.67 [95% CI=1.84–3.51; *p* <0.001]; Fig. 3B ). We did not find any significant heterogeneity between the two-subgroup comparisons.

### Effects of Chlorhexidine on the Viral Load

**Fig. 4:**
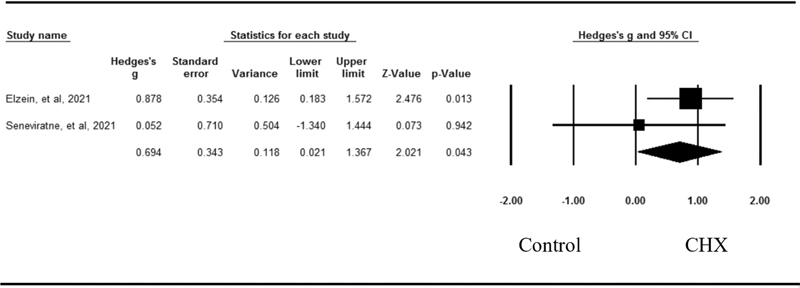
Forest plot of mean effect sizes for SARS-CoV-2 mean Ct value between CHX group compare with control group. Heterogeneity: *Q* =0.87, *p* =0.35, *I* ^2^ =0.00. CHX, chlorhexidine; CI, confidence interval; Ct, cycle threshold; SARS-CoV-2, Severe acute respiratory syndrome coronavirus 2.

Two studies compared the immediate pos-treatment effect of CHX on the viral load to a control in patients with COVID-19 ( Fig. 4 ). CHX had a pooled effect size of 0.69 (95% CI=0.02–1.37; *p* =0.04) for reducing the viral load. There was no evidence of heterogeneity across these studies ( *Q* =0.87, *p* =0.35, *I* ^2^ =0.00).

### Effects of Povidone-Iodine on the Viral Load

**Fig. 5:**
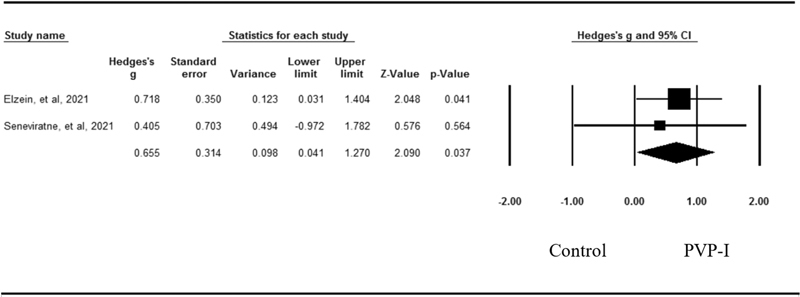
Forest plot of mean effect sizes for SARS-CoV-2 mean Ct value between PVP-I group compare with control group. Heterogeneity: *Q* =0.06, *p* =0.81, *I* ^2^ =0.00. CI, confidence interval; Ct, cycle threshold; PVP-I, povidone-iodine; SARS-CoV-2, Severe acute respiratory syndrome coronavirus 2.

As presented in Fig. 5 , two studies compared the posttreatment effect of PVP-I on the viral load to a control in patients with COVID-19. The pooled effect size of PVP-I was 0.66 (95% CI=0.04–1.27; *p* =0.04) for decreasing the viral load. No evidence of heterogeneity was observed across these studies ( *Q* =0.06, *p* =0.81, *I* ^2^ =0.00).

### Risk of Bias of the Included Studies

The result of the risk of bias assessment is described in Supplementary Table S3 (available in the online version) for RCTs. All 10 studies addressed concerns about sufficient randomization and incomplete outcome data. Only eight studies adequately concealed allocation. Participants and personnel were blind to treatment assignment in six studies, whereas assessors were unaware in five studies.

## Discussion

Both CHX and PVP-I had a significant effect to reduce the number of negative RT-PCR results compared with the control group. Similarly, CHX and PVP-I also gained similar moderate effect size on reducing viral load in patients with COVID-19 compared to a control. The current study employed a rigorous methodology with a general low risk of bias; consequently, the results should be regarded to have a high degree of confidence.

Antiseptic mouthwashes have long been used as a common preprocedural modality prior to some conventional dental treatments, especially surgeries. 39 They are crucial for reducing the counts of infectious bacteria and other microorganisms inside the oral cavity. 16 According to recent studies, cleansing the oral cavity can help manage and lower the likelihood of SARS-CoV-2 transmission. 40

CHX is a broad-spectrum antiseptic that causes bacterial cell wall lysis in gram-positive and gram-negative bacteria, aerobes, facultative anaerobes, and fungi by increasing bacterial cell wall permeability. 41 42 In dentistry, it is used to treat periodontal disease by decreasing bacteria counts in dental plaque. 43 CHX at 0.12% has produced Ct values of 10.5±0.5 and 11±1.0 after 30 and 60seconds of exposure, respectively, compared to Ct values of 9.5±0.5 and 11±2, respectively, for 1% PVP-I, indicating the efficacy of both mouthwashes against SARS-CoV-2. 44 However, another *in vitro* study using a mouthwash containing 0.12% CHX gluconate (0.12%) in combination with 0.05% cetylpyridinium chloride as an antiseptic failed to inactivate SARS-CoV-2 sufficiently after 30seconds. 45 Despite these findings, the authors acknowledged that CHX could reduce the viral load in the mouth based on its persisting effects opposed to short-term treatment with antiseptics *.*

PVP-I is a water-soluble iodine compound that has long been used as a skin antiseptic and mouthwash. After free iodine dissociates from polyvinylpyrrolidone, it quickly enters microorganisms and destroys them by disrupting proteins and oxidizing nucleic acid structures. PVP-I is safe, and it does not cause tooth or tongue discoloration or taste disturbance. 46 PVP-I was proven in previous investigations to have strong virucidal activity. The use of 1% PVP-I and 0.2% CHX mouthwash increased the mean Ct value more strongly than distilled water after 30seconds of rinsing in patients infected with SARS-CoV-2. 33 These results agree with those of Jain et al. 44

CHX can have adverse effects if used for 4 weeks or longer. 47 Specifically, CHX mouthwash causes brown discoloration on the surface of the teeth which may be removed by a dental expert after scaling and polishing. 48 However, CHX mouthwash causes no or minimal discoloration after 1 or 2 weeks of treatment. 49 Other side effects of CHX include taste disruption and mouth lining pain, both of which are temporary and normally reversible after mouthwash use is stopped. 4 Conversely, long-term PVP-I mouthwash use does not result in discoloration. Oral formulations of PVP-I remain popular because of their wide range of effects and tolerability. 46 Considering the safety for long-term usage, hence, we recommend PVP-I to use in dental care setting.

## Limitations

To ensure high internal validity, this study included experimental design evaluating the effects of CHX or PVP-I on the viral load in patients with COVID-19. However, several limitations must be considered. First, this study included a relatively small number of studies with small sample sizes. Second, the patient population, outcome measurements, and treatment duration used in the included studies varied, and no data on the protocol that maximizes patient safety in the dental office to avoid exposure to aerosolized particles among dental personnel and other people visiting the office ( Table 2 ).

## Conclusion

This review revealed that CHX and PVP-I had significant effects on reducing the number of negative RT-PCR results compared with the control group among COVID-19 patients. Even though both CHX and PVP-I were having similar efficacy, however, for long-term usage, PVP-I seems to be safer. Additional studies using adequate randomization methods and larger sample sizes are required.

## Highlights

- This review revealed that CHX and PVP-I had significant effects on reducing the number of negative RT-PCR results compared with the control group among COVID-19 patients.
- Even though both CHX and PVP-I were having similar efficacy, however, for long-term usage, PVP-I seems to be safer.
- To ensure high internal validity, this study included experimental design evaluating the effects of CHX or PVP-I on the viral load in patients with COVID-19.
