# Supplementary material for: Effects of Chlorhexidine and Povidone-Iodine on the SARS-CoV-2 Load: A Systematic Review and Meta-analysis
Source: Eur J Dent. 2022 Sep 8;17(3):587–601. doi: 10.1055/s-0042-1753470 (PMC10569867; doi:10.1055/s-0042-1753470)
Supplement: Supplementary file 1 — Supplementary Material [file 10-1055-s-0042-1753470-s2242074.pdf]

Supplementary Table S1 Search strategy

Database	Keywords
PubMed	(("Oral rinses"[All Fields] OR "mouth rinses"[All Fields] OR "Oral Mouth Rinses"[All Fields] OR "Mouth Wash"[All Fields] OR "mouthwashes"[All Fields] OR "mouthwash"[All Fields] OR "mouthwashing"[All Fields]) AND ("Viral Load"[All Fields] OR "Viral Burden"[All Fields] OR "viral inactivation"[All Fields] OR "Virus Inactivation"[All Fields]) AND ("covid 19"[All Fields] OR "sars cov 2"[All Fields] OR "severe acute respiratory syndrome coronavirus 2"[All Fields] OR "coronavirus"[All Fields] OR "covid 19"[All Fields] OR "Coronavirus disease 2019"[All Fields]))
EMBASE	("oral rinses" OR "mouth rinses"/exp OR "mouth rinses" OR "oral mouth rinses" OR "mouth wash"/exp OR "mouth wash" OR "mouthwashes"/exp OR "mouthwashes" OR "mouthwash"/exp OR "mouthwash" OR "mouthwashing"/exp OR "mouthwashing") AND ("viral load"/exp OR "viral load" OR "viral burden"/exp OR "viral burden" OR "viral inactivation" OR "virus inactivation"/exp OR "virus inactivation") AND ("covid-19"/exp OR "covid-19" OR "sars cov 2"/exp OR "sars cov 2" OR "severe acute respiratory syndrome coronavirus 2"/exp OR "severe acute respiratory syndrome coronavirus 2" OR "coronavirus"/exp OR "coronavirus" OR "covid 19"/exp OR "covid 19" OR "coronavirus disease 2019"/exp OR "coronavirus disease 2019")
Cochrane Library	("Oral rinses" OR "mouth rinses" OR "Oral Mouth Rinses" OR "Mouth Wash" OR "mouthwashes" OR "mouthwash" OR "mouthwashing") AND ("Viral Load" OR "Viral Burden" OR "viral inactivation" OR "Virus Inactivation") AND ("COVID-19" OR "sars cov 2" OR "severe acute respiratory syndrome coronavirus 2" OR "coronavirus" OR "COVID 19" OR "Coronavirus disease 2019") in Title Abstract Keyword - (Word variations have been searched)
ProQuest	("Oral rinses" OR "mouth rinses" OR "Oral Mouth Rinses" OR "Mouth Wash" OR "mouthwashes" OR "mouthwash" OR "mouthwashing") AND ("Viral Load" OR "Viral Burden" OR "viral inactivation" OR "Virus Inactivation") AND ("COVID-19" OR "sars cov 2" OR "severe acute respiratory syndrome coronavirus 2" OR "coronavirus" OR "COVID 19" OR "Coronavirus disease 2019")

Supplementary Table S2 List of the excluded studies after a full-text review

Author (year)	Reasons of exclusion
Babady et al (2021)[1]	Did not provide data of interest
Chaudhary et al (2021)[2]	Did not provide data of interest
Khan et al (2020)[3]	Did not provide data of interest

Supplementary Table S3 Risk of bias RoB 1.0

Study (year)	Random sequence generation	Allocation concealment	Blinding of participants and personnel	Blinding of outcome assessment	Incomplete outcome data addressed
Avhad et al (2020)[29]	L	U	L	L	L
Choudhury et al (2021)[30]	L	U	U	U	L
Costa et al (2021)[31]	L	L	L	L	L
Eduardo et al (2021)[32]	L	L	L	L	L
Elzein et al (2021)[33]	L	L	L	L	L
Guenezan et al (2021)[34]	L	L	H	U	L
Huang et al (2021)[35]	L	L	L	L	L
Mohamed et al (2020)[36]	L	L	H	U	L
Mukhtar et al (2021)[37]	L	L	H	U	L
Seneviratne et al (2021)[38]	L	L	L	U	L

Abbreviations: H, high risk; L, low risk; U, unclear risk of bias.

 © 2022. The Author(s).

Conflict of Interest
None declared.

References

1 Babady NE, McMillen T, Jani K, et al. Performance of severe acute respiratory syndrome coronavirus 2 real-time rt-pcr tests on oral rinses and saliva samples. J Mol Diagn 2021;23(01):3–9

2 Chaudhary P, Melkonyan A, Meethil A, et al. Estimating salivary carriage of severe acute respiratory syndrome coronavirus 2 in nonsymptomatic people and efficacy of mouthrinse in reducing viral load: a randomized controlled trial. J Am Dent Assoc 2021; 152(11):903–908

3 Khan MM, Parab SR, Paranjape M. Repurposing 0.5% povidone iodine solution in otorhinolaryngology practice in Covid 19 pandemic. Am J Otolaryngol 2020;41(05):102618

 © 2022. The Author(s).
